# Evaluating biocontrol potential of 6 parasitoid species (Hymenoptera) on apple and cherry aphids (Hemiptera: Aphididae) using no-choice bioassays

**DOI:** 10.1093/jisesa/ieag023

**Published:** 2026-03-15

**Authors:** Rachele Quaglino, Nina Haas, Maiara Bastos Arteca, Leslie McCluckie, Barbara Egger

**Affiliations:** Fruit Production Extension, Department of Plants and Plant Products, Agroscope, Wädenswil, Switzerland; Fruit Production Extension, Department of Plants and Plant Products, Agroscope, Wädenswil, Switzerland; Fruit Production Extension, Department of Plants and Plant Products, Agroscope, Wädenswil, Switzerland; Fruit Production Extension, Department of Plants and Plant Products, Agroscope, Wädenswil, Switzerland

**Keywords:** natural enemy, orchard, Aphelinidae, Braconidae, host suitability

## Abstract

European apple and cherry production depend on plant protection substances, but the variety of available options is continuously declining. Consequently, there is a need for alternative pest control strategies in orchards. An alternative method is the use of biocontrol agents, such as parasitoids. A range of parasitoid species is commercially available and a well-recognized strategy against aphid pests in protected crops. However, the efficacy of these species against apple and cherry aphid pests is still unexplored. Therefore, we conducted no-choice bioassays to assess the compatibility of 6 parasitoid species—*Aphelinus abdominalis* Dalman (Hymenoptera: Aphelinidae), *Aphidius colemani* Viereck (Hymenoptera: Braconidae), *Aphidius ervi* Haliday (Hymenoptera: Braconidae), *Aphidius matricariae* Haliday (Hymenoptera: Braconidae), *Ephedrus cerasicola* Stary (Hymenoptera: Braconidae), and *Praon volucre* Haliday (Hymenoptera: Braconidae)—against 3 target aphid species: *Myzus cerasi* Fabricius (Hemiptera: Aphididae), *Aphis pomi* De Geer (Hemiptera: Aphididae), and *Dysaphis plantaginea* Passerini (Hemiptera: Aphididae). The assays were conducted under greenhouse conditions on host tree cuttings (*M. cerasi*) or under controlled climate chamber conditions on saplings (*A. pomi, D. plantaginea*) with laboratory-reared aphid colonies. For each parasitoid species, the aphid colonies were inoculated with 5 to 10 females for a minimum of 72 h. The resulting parasitism rate was evaluated after 14 d. Our experiments show that host compatibility varies considerably between aphid species. Among the parasitoids tested, *P. volucre* and *Aphi. matricariae* emerged as the most consistent and effective species across all hosts. This suggests ecological compatibility and indicates that these 2 species are promising candidates for biological control in fruit orchards.

## Introduction

In European pome and stone-fruit production, insect pests pose a major threat, directly damaging crops and reducing both fruit quality and yield (Lamichhane et al. 2015, Helvacı 2022). Pests are estimated to cause 25% to 40% yield losses globally in crop production systems (FAO 2026), and losses can exceed 50% in untreated apple orchards (Bapfubusa et al. 2025). Among the most damaging pests in European orchards are leaf aphids, sap-feeding insects (McLaren and Fraser 2002, Blommers et al. 2004, Stoeckli et al. 2008). The apple aphid *Aphis pomi* (De Geer, Hemiptera: Aphididae) is widespread across Europe, North America, and the Middle East and can reduce tree growth and productivity, particularly in young apple trees (*Malus domestica* Borkhausen, Rosales: Rosaceae) (Arbab et al. 2006). The rosy apple aphid *Dysaphis plantaginea* Passerini (Hemiptera: Aphididae) is considered one of the most harmful apple pests in Europe, causing leaf curling, shoot deformation, and fruit distortion, with yield losses of up to 80% in untreated orchards (Blommers et al. 2004, Dib et al. 2016). The black cherry aphid *Myzus cerasi* Fabricius (Hemiptera: Aphididae) is the dominant aphid pest on cherry trees (*Prunus avium* Linnaeus, Rosales: Rosaceae), damaging young leaves, reducing photosynthesis, and impairing fruit quality (McLaren and Fraser 2002). Effective control of these aphids is critical for maintaining healthy and productive orchards.

Synthetic pesticides have been the primary tool to control aphids in orchards (Barzman et al. 2015). While effective in the short term, their use has severe drawbacks: they reduce biodiversity by killing nontarget organisms and weaken natural enemy populations that contribute to pest regulation (Mesnage and Séralini 2018, Elhamalawy et al. 2024). As a result, pesticide use is increasingly restricted across Europe, intensifying the need for alternative solutions that maintain orchard productivity while reducing chemical inputs.

Biological control represents one of the most promising alternatives for aphid control. It relies on the action of natural enemies—predators, parasitoids, and pathogens—to regulate pest populations (Landis et al. 2000, Colmenarez and Vasquez 2024). Among potential biocontrol agents, hymenopteran parasitoids are particularly effective due to their high host specificity and close coevolution with aphids (Godfray 2016, Kishinevsky and Ives 2024). This specificity allows parasitoids to track aphid populations closely while minimizing risks to nontarget species. Subfamilies such as the Aphidiinae (eg *Aphidius*, *Ephedrus*, and *Praon* genera) and the family Aphelinidae (eg *Aphelinus* genus) are widely used in pest control and play a crucial role in agriculture and forestry (Wei et al. 2005, Völkl et al. 2007, Gadallah et al. 2022). Their effectiveness has been particularly well demonstrated in greenhouse systems, where parasitoid releases are a standard component of integrated pest management against aphids and mites (Boivin et al. 2012, Hance et al. 2017). Extending such approaches to an open-field system, like orchards, could be a promising next step but requires robust evidence of host-parasitoid compatibility under orchard-relevant conditions.

Although aphid-parasitoid interactions in orchard systems have been studied, existing research has focused on a limited number of aphid and parasitoid species. For the apple aphid *A. pomi*, most documented parasitoids belong to the Braconidae family, particularly Aphidiinae species such as several *Praon* spp. (Carroll and Hoyt 1986), *Binodoxys angelicae* (Haliday, Hymenoptera: Aphidiinae) as well as Hymenoptera: Aphelinidae species like *Aphelinus paramali* (Zehavi and Rosen), which has been reported parasitizing *A. pomi* in the field and under laboratory conditions (Darsouei et al. 2011). Similarly, the rosy apple aphid *D. plantaginea* is known to be parasitized by Aphidiinae species including *Aphidius*, *Binodoxys*, and *Ephedrus* spp. (Peusens et al. 2006, Tougeron et al. 2023). In contrast, the black cherry aphid *M. cerasi* remains comparatively understudied; most parasitoid research has focused on its close relative *Myzus persicae* Sulzer (Hemiptera: Aphididae), with *M. cerasi* itself only reported to interact with *Ephedrus* species in food-web studies (Alhmedi et al. 2023).

To address this gap, we evaluated the potential of 6 species of parasitoid wasps, available in the commercial parasitoid mixture, Fresa-Protect (Viridaxis SA, Belgium), which contains: *Aphelinus abdominalis* Dalman (Hymenoptera: Aphelinidae), *Aphidius colemani* Viereck (Hymenoptera: Aphidiinae), *Aphidius ervi* Haliday (Hymenoptera: Aphidiinae), *Aphidius matricariae* Haliday (Hymenoptera: Aphidiinae), *Ephedrus cerasicola* Stary (Hymenoptera: Aphidiinae), and *Praon volucre* Haliday (Hymenoptera: Aphidiinae). While these parasitoids are used for aphid control in strawberry production in plastic tunnels, their effectiveness against orchard aphid pests remains largely unexplored. Because the performance of both aphids and parasitoids is strongly temperature-dependent, we conducted controlled laboratory assays at temperatures representing optimal performance conditions. We quantified the parasitism success of all 6 species on the 3 major aphids of European pome and stone fruit—*A. pomi*, *D. plantaginea*, and *M. cerasi*—to identify which parasitoids can successfully develop on these hosts and thus show promise as biological control agents in orchard systems.

## Materials and Methods

### Assay Species and Preparation

**Fig. 1.**
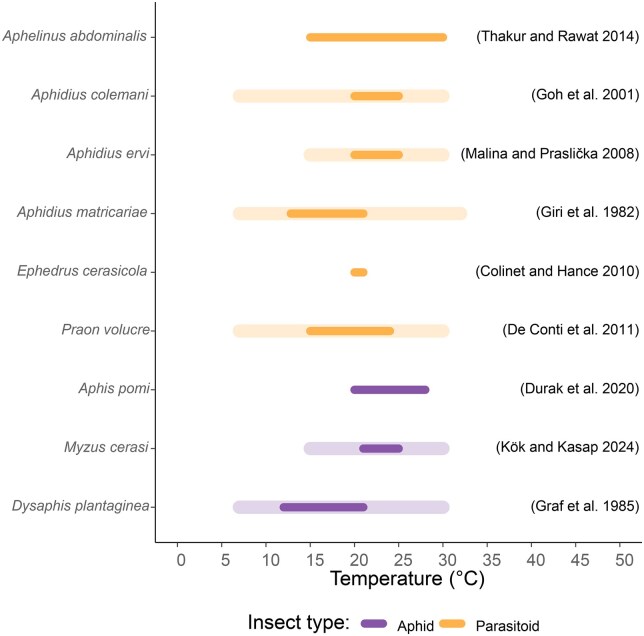
Thermal tolerance of parasitoid (orange) and aphid (purple) species. Bars show the range of temperature at which the species survive (light pastel color) and show optimal performance (darker color). The data used for this figure are cited in the figure and the text.

Six parasitoid wasp species—*Aphe. abdominalis, Aphi. colemani, Aphi. ervi, Aphi. matricariae, E. cerasicola*, and *P. volucre—*supplied by Viridaxis SA (Belgium) in the commercial product Fresa-Protect were evaluated for their ability to parasitize *M. cerasi* (cherry), *A. pomi* (apple), and *D. plantaginea* (apple). Detailed breeding conditions of the parasitoid species are confidential and cannot be disclosed here. Parasitoids were delivered as mummified aphids in ventilated cardboard boxes (4 cm diameter, 10 cm height). Upon delivery, parasitoids were transferred for hatching and mating to ventilated containers (14 cm diameter, 35 cm height) at 25 °C, the temperature selected considering the optimal developmental temperature range of most of the tested parasitoid species (Fig. 1). Mating was facilitated for 48 h under the provision of pure honey and water in separate 1.5 ml plastic tubes (Eppendorf SE, Germany).

Colonies of *M. cerasi* were established from field-collected individuals at the Agroscope experimental farm Breitenhof (Steinobstzentrum Breitenhof, Wintersingen, Switzerland, 47°29′48.1″N 7°48′46.2″E, spring 2024). Aphids were reared on 1-year-old cherry saplings in a climate chamber (22 °C, 60% relative humidity [RH], 16:8 photoperiod). Individuals of *A. pomi* were collected in an apple nursery in Sandhof (Wädenswil, Switzerland, 47°13′19.5″N 8°40′12.0″E, on 23 July 2024) and reared in a climate chamber (22 °C, 70% RH, 16:8 photoperiod) on apple saplings. Colonies of *D. plantaginea* were initiated from individuals collected in an apple plantation in Gottshalden (Horgen, Switzerland, 47°14′50.5″N 8°37′42.3″E, on 15 May 2025). Aphids were reared on apple saplings in a climate chamber (22°C, 70% RH, 16:8 photoperiod).

### Assay Temperature

To select suitable environmental conditions for the assays, we compiled information on the thermal performance of all aphid and parasitoid species involved (Fig. 1). Parasitoids show overlapping optimal temperature ranges: *Aphe. abdominalis* is most active between 15 and 30 °C (Thakur and Rawat 2014), *Aphi. colemani* performs best at 20 to 25 °C but can tolerate 7 to 30 °C (Goh et al. 2001); and *Aphi. ervi* exhibits sufficient activity between 15 and 30 °C (Malina and Praslička 2008). *E. cerasicola* performs best near 21 °C (Colinet and Hance 2010), while *Aphi. matricariae* has an optimal range of 12.8 to 21 °C and can develop from 7 to 32 °C (Giri et al. 1982). *P. volucre* shows highest parasitism between 15 and 24 °C and remains active from 7 to 30 °C (De Conti et al. 2011, Farhad et al. 2011). Published developmental temperatures of the leaf aphids were likewise consistent with moderate temperature conditions: *M. cerasi* performs well between 15 and 25 °C (Özder and Tayat 2019, Kök and Kasap 2024), *A. pomi* at 20 to 28 °C (Durak et al. 2020), while *D. plantaginea* develops optimally at cooler temperatures between 7 and 22 °C (Graf et al. 1985).

Given these ranges, a temperature of 22 °C and 70% RH were selected for all climate chamber experiments (*A. pomi, D. plantaginea*), while temperature and RH under greenhouse conditions (*M. cerasi*) could not be fully controlled, but was constantly monitored (Supplementary Fig. S1). The chosen temperature maximizes overlap among the parasitoid species, remains within or close to the documented performance window of all aphids and ensures consistent and comparable assay conditions across hosts and parasitoids.

### Experimental Design

Between spring 2024 and summer 2025, 3 no-choice host compatibility assays were carried out in the greenhouse or in climate chambers, respectively. Each focused on a single aphid species. The aphid colonies were exposed to each of the 6 parasitoid species separately, along with a negative control where no parasitoid exposure occurred, resulting in a total of 7 treatments. Long exposure durations of 72 h (*M. cerasi, A. pomi*) and 14 d (*D. plantaginea*) were chosen to enhance the probability of parasitism, since information about host suitability of the tested aphid species was lacking.

The number of replicates depended on the availability of host trees and varied for each experiment: for *M. cerasi* 8 replicates per treatment were carried out and for *A. pomi* and *D. plantaginea* 6 replicates per treatment were initiated.

### Parasitism Bioassay *M. cerasi*

The bioassays of *M. cerasi* parasitism were done on a total of 56 freshly cut cherry shoots (7 treatments×8 replicates). Each shoot was stabilized by floral foam (Oasis Floral Products) in ventilated acrylic cylinders (15 cm diameter, 25 cm height) with water reservoirs. The cylinders were covered with chiffon fabric to prevent contamination and escape of the studied insects. A protective polystyrene barrier together with nonwoven fabric above the floral foam prevented aphids and parasitoids from falling into the water. Water levels were monitored and refilled as needed.

The shoots were kept in a greenhouse under natural light, where temperatures ranged between 20–45 °C and 14% to 55% RH. Temperature and RH were monitored with SHT43 DemoBoard sensors (Sensirion AG, Switzerland). Temperature peaks occurred due to failure of the ventilation system and were of short duration (Supplementary Fig. S1).

Each shoot was first inoculated with 30 adult *M. cerasi* to establish fresh nymph colonies. After 72 h, the adult aphids were removed, leaving nymph populations of 21 to 159 per shoot for the parasitism assays. Next, 5 female parasitoids per replicate were introduced into the cylinders. The sex was determined visually by the presence of an ovipositor. *Aphe. abdominalis* sexing was unreliable, therefore 10 unsexed individuals were introduced per replicate to ensure sufficient female presence. Parasitoids were removed from the colonies with an aspirator after 72 h of exposure.

### Parasitism Bioassay *A. pomi*

For the assay of *A. pomi*, 37 apple saplings (6 treatments×6 replicates + 1 control) were placed in individual plastic trays in enclosed mesh cages (BugDorm, 47.5 × 47.5 × 47.5 cm) on metallic racks in a climate chamber (22 °C, 70% RH, 16:8 photoperiod). In this bioassay, only 1 negative control replication could be conducted due to restrictions in plant availability. The saplings were watered every 3 d. To establish clean aphid colonies, either 5 adults or 30 nymphs were inoculated on each sapling. The adult aphids were removed after 4 d when colonies reached 30 to 40 nymphs per sapling; next, parasitoids were introduced into the BugDorms. In each replicate, 5 females were added; sex was determined by the presence of an ovipositor. *Aphe. abdominalis* sexing was unreliable, therefore 10 unsexed individuals were introduced per replicate to ensure sufficient female presence. Parasitoids were removed from the colonies with an aspirator after 72 h of exposure.

### Parasitism Bioassay *D. plantaginea*

For the assay of *D. plantaginea*, 42 apple saplings (7 treatments × 6 replicates) were placed in the BugDorms on metallic racks in the climate chambers (22 °C, 70% RH, 16:8 photoperiod). The trees were watered as regularly as needed. Five adult aphids were added to each tree and removed after 5 d to establish fresh nymph colonies. The nymph colony size was standardized at 25 to 50 individuals per sapling by transferring additional nymphs when necessary. Next, parasitoids were introduced into the BugDorms. In each replicate, 5 females were added; sex was determined visually by the presence of an ovipositor. *Aphe. abdominalis* sexing was unreliable, therefore 10 unsexed individuals were introduced per replicate to ensure sufficient female presence. Parasitoids were left until natural death to ensure adequate exposure.

### Monitoring and Data Collection

Temperature, host-plant health, and water levels were monitored and adjusted throughout the incubation period. The development time from oviposition to adult emergence was approximately 17 d for *Aphidius* spp. and *E. cerasicola*, 21 d for *P. volucre* and 25 d for *Aphe. abdominalis*. To avoid interference from a second parasitoid generation (emergence, mating, and further parasitism), all assays were terminated after 14 d. At this time, the number of mummies, live aphids, and dead aphids was recorded. Mummies were identified visually using documented species-specific appearance (Supplementary Fig. S2). In addition, overall tree or shoot health was visually assessed on a scale from 1 (poor condition) to 5 (optimal condition) (Supplementary Fig. S3).

### Statistical Analysis

All statistical analyses were conducted in R (version 4.5.1) (Team RC 2023). The data were processed and visualized with the packages “dplyr” (Wickham et al. 2026) and “ggplot2” (Wickham 2016), and models were fitted using “glmmTMB” (Brooks et al. 2017). For each experiment (*M. cerasi*, *A. pomi*, and *D. plantaginea*), parasitism rate was calculated as the proportion of aphids mummified relative to the total number of aphids (alive, dead, and mummified).


$$\mathrm{Parasitism}\;\mathrm{rate}=\frac{N_{\mathrm{mummies}}}{N_{\mathrm{alive}\;\mathrm{aphids}}+N_{\mathrm{dead}\;\mathrm{aphids}}+N_{\mathrm{mummies}}}$$

In addition, aphid survival, aphid mortality, and host tree health were derived as secondary response variables.

To assess differences in parasitism rate among treatments, generalized linear models were fitted with a beta-binomial error distribution to handle overdispersion. Zero-inflation was also tested and included, when treatments were expected to show many zeros. Parasitism rates in the negative control treatments were expected to be zero and were excluded from the analysis. The overall significance of the treatment effect was evaluated using type III Wald χ^2^ tests with the function “Anova” in the “car” package (Fox et al. 2024). Model assumptions were verified by simulation-based residual diagnostics using the “DHARMa” package (Hartig 2024). Post hoc pairwise comparisons among treatments were conducted with estimated marginal means using “emmeans” (Lenth et al. 2025), and significance letters were assigned with “multcompView” after Tukey adjustment for multiple testing (Graves et al. 2024).

Replication varied slightly among experiments (*M. cerasi *= 8 replicates, *A. pomi *= 6 replicates, *D. plantaginea *= 6 replicates), due to host-plant availability.

Establishing consistent conditions for all plant-aphid-parasitoid combinations was difficult, due to host-plant fitness and ventilation failures (Supplementary Fig. S3). This led to high aphid mortality rates, especially for *M. cerasi*, unrelated to the parasitism treatments (Supplementary Fig. S4). All procedures and data were documented transparently to support reproducibility.

## Results

**Fig. 2.**
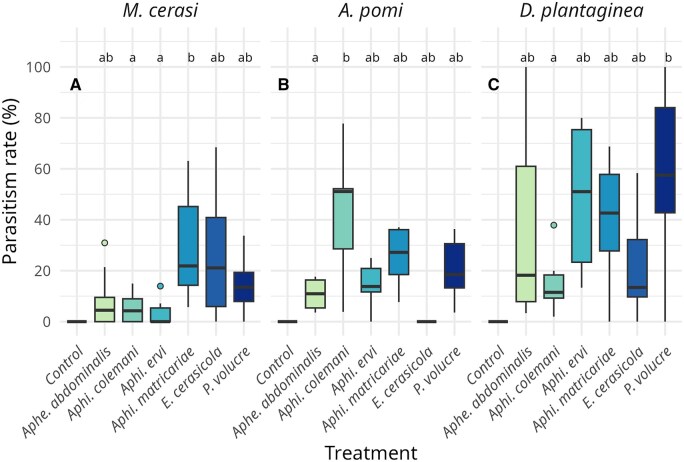
Boxplot illustrating parasitism rate (%) of the different parasitoid species. Boxes represent the distributions of parasitism rates of the treatments and points depict outliers. Different letters show significant differences between treatments within 1 aphid species (*P* <0.05). A) Percentage of parasitism on *Myzus cerasi*. B) Percentage of parasitism on *Aphis pomi*. C) Percentage of parasitism on *Dysaphis plantaginea*.

We evaluated the parasitism success of 6 parasitoid species on 3 aphid hosts. A summary of the parasitism rates for each parasitoid and aphid species is given in Table 1. For all 3 aphid species, treatment had a significant effect on parasitism rate, highlighting performance differences among parasitoid species (type III Wald χ^2^ tests, *M. cerasi: χ*^2^ = 20.68, *df* = 5, *P* = 0.001; *A. pomi: χ*^2^ = 15.55, *df* = 5, *P* = 0.008; *D. plantaginea: χ*^2^ = 12.53, *df* = 5, *P* = 0.03; Fig. 2). Raw aphid counts (*N*_alive aphids_, *N*_dead aphids_, *N*_mummies_) can be found in Supplementary Figs. S4 to S6 and Supplementary Table S1.

**Table 1.** Mean parasitism rate (%) and SE of parasitoids toward host species

Experiment	Treatment	Average parasitism rate (%)	SE
***Myzus cerasi***	Control	0.0	0.0
	*Aphelinus abdominalis*	8.4	4.1
	*Aphidius colemani*	5.5	2.1
	*Aphidius ervi*	3.2	1.8
	*Aphidius matracariae*	28.9	7.1
	*Ephedrus cerasicola*	26.8	8.9
	*Praon volucre*	14.3	3.7
***Aphis pomi***	Control	0.0	*0.0*
	*Aphelinus abdominalis*	10.8	2.6
	*Aphidius colemani*	42.9	10.7
	*Aphidius ervi*	14.5	3.7
	*Aphidius matracariae*	25.6	4.9
	*Ephedrus cerasicola*	0.0	0.0
	*Praon volucre*	20.5	5.2
***Dysaphis plantaginea***	Control	0.0	0.0
	*Aphelinus abdominalis*	36.6	16.4
	*Aphidius colemani*	15.3	5.1
	*Aphidius ervi*	48.8	12.3
	*Aphidius matracariae*	40.0	10.4
	*Ephedrus cerasicola*	22.0	8.9
	*Praon volucre*	57.6	14.8

### Parasitism Bioassay *M. cerasi*

Mummies were recovered from all 6 parasitoid treatments, confirming that each species successfully parasitized *M. cerasi* in the no-choice assay. No mummified aphids were observed in the negative control. Although parasitism rates differed among species, the pairwise differences were not statistically significant (all *P* values less than 0.05; Fig. 2A). *Aphi. matricariae* achieved the highest rate (≈29%), closely followed by *E. cerasicola* (≈27%) and *P. volucre* (≈14%). In contrast, *Aphi. colemani, Aphi. ervi*, and *Aphe. abdominalis* showed low host compatibility, with parasitism below 10%.

### Parasitism Bioassay *A. pomi*

Parasitism was observed for all species except *E. cerasicola*. No mummies occurred in the negative control. Overall, species differences were modest (Fig. 2B), though *Aphi. colemani* clearly outperformed the others, parasitizing ≈43% of the host individuals. In contrast, *Aphe. abdominalis* showed a significantly lower rate (10.8%; *P* = 0.006). The remaining species *(Aphi. ervi, Aphi. matricariae, P*. *volucre*) exhibited intermediate success, with parasitism ranging between 15% and –25%.

### Parasitism Bioassay *D. plantaginea*

All parasitoid species successfully parasitized *D. plantaginea*, while no mummies were found in the negative control. Pairwise differences among treatments were again mostly nonsignificant (*P* > 0.05; Fig. 2C). *P. volucre* performed best, parasitizing over half of the aphid colony, whereas *Aphi. colemani* showed significantly lower success (15.3%; *P* = 0.02). *E. cerasicola* and *Aphe. abdominalis* achieved medium rates (20% to 40%), while *Aphi. ervi* and *Aphi. matricariae* maintained high compatibility, parasitizing 40% to 50% of aphids.

## Discussion

This study provides a systematic evaluation of 6 commercially available parasitoid species against 6 major aphid pests of European orchards under controlled laboratory or greenhouse conditions, respectively. By testing all host-parasitoid combinations, we offer an initial assessment of ecological compatibility and identify which species may hold promise for future biological control applications in fruit production systems.

Across host species, parasitism success varied markedly, suggesting that host suitability is strongly species-specific. *A. pomi* was readily parasitized by *Aphi. colemani*, *Aphi. matricariae*, and *P. volucre*, consistent with earlier work showing that *Aphis* spp. are generally acceptable hosts for several Aphidiinae parasitoids. Examples are *Praon unicum* parasitizing *A. pomi*, or *Aphi. colemani* parasitizing *Aphis gossypii* (Carroll and Hoyt 1986, Ode et al. 2005, Al Hadi and Mansor 2025). The failure of *E. cerasicola* to parasitize *A. pomi* contrasts with the successful parasitism reported for closely related *Ephedrus* species: *Ephedrus persicae* and *Ephedrus plagiator* were observed parasitizing *M. cerasi* (Alhmedi et al. 2018). Our resluts suggest a more restricted host range for *E. cerasicola* than previously assumed (Tougeron et al. 2023). For *D. plantaginea*, all parasitoids were capable of parasitism, with particularly high performance by *P. volucre*, *Aphi. matricariae*, and *Aphi. ervi*. These results align with findings from earlier studies reporting efficient parasitism of *D. plantaginea* by *Aphi. matricariae, Aphi. Ervi*, and *E. cerasicola*, but offer novel insights into the specific compatibility of the other 3 parasitoid species (Alhmedi et al. 2018, Tougeron et al. 2021). The broad parasitism spectrum observed in this host suggests that *D. plantaginea* may be accessible to a relatively wide range of parasitoid species in orchard systems. Also *M. cerasi* was parasitized by all 6 parasitoid species, indicating ecological compatibility, yet interpretation for this host is limited by the high mortality observed in all experimental units, including the negative control (Supplementary Fig. S4). This mortality likely reflects methodological constraints, including declining shoot quality and temperature fluctuations during incubation, both of which are known to negatively affect *M. cerasi* survival (Kök and Kasap 2024). As a result, comparisons among treatments should be viewed cautiously, and future work would benefit from improved rearing substrates or reduced experimental duration to maintain stable host conditions. The observed host-specific parasitism patterns likely reflect differences in coevolutionary history and physiological compatibility between aphids and parasitoids (Gimmi and Vorburger 2024, Henry et al. 2025). Aphid defensive traits, including symbiont-mediated resistance and immune responses, are also known to modulate parasitoid success (Gagic et al. 2016, Vorburger 2018). In addition, ecological factors such as host-plant associations and microclimatic conditions may further shape parasitoid-host interactions (Alhmedi et al. 2023, Nguyen et al. 2023).

The thermal requirements of aphids and parasitoids strongly affect their interactions and determines whether biological control agents can be effective under field conditions. All 6 parasitoid species tested operate within temperature ranges commonly recorded in European orchards during spring and summer (8 to 28 °C; Begert and Frei 2018). This thermal overlap suggests that the species demonstrating high parasitism in laboratory assays could also be active during periods of aphid population growth in the field. Nevertheless, laboratory temperatures are constant, whereas field conditions fluctuate across diel and seasonal cycles. Such variation can influence parasitoid development time, longevity, and fecundity, as well as aphid reproductive rates (Roux et al. 2010, Pan et al. 2024). Therefore, thermal compatibility observed in the laboratory should be interpreted as a necessary but not sufficient condition for effective field performance (Sigsgaard 2002).

Among the parasitoids examined, *P. volucre* and *Aphi. matricariae* showed the most consistently high parasitism across all 3 hosts. Both species are known for broad host ranges and tolerance of variable environmental conditions (Stilmant et al. 2008, Boivin et al. 2012, Hance et al. 2017), which may explain their robust performance in our assays. Their generalist traits suggest that they could persist under the diverse microclimatic and ecological conditions found in orchards, making them promising candidates for future field evaluation (Giri et al. 1982, De Conti et al. 2011). Conversely, the lower parasitism success of *E. cerasicola* and *Aphe. abdominalis* across hosts may indicate more specialized host requirements or narrower ecological niches, which could limit their applicability in orchard biocontrol programs.

Although this study provides valuable first insights into potential parasitoid-host associations relevant to orchard pest management, several methodological limitations should be acknowledged. Negative control replicates could not be completed in 1 experiment due to lack of apple saplings, and challenges associated with maintaining consistent plant quality led to variability in host availability and survival. Additionally, no-choice assays, while essential for establishing fundamental host compatibility, do not capture the behavioral complexity of parasitoid decision-making in heterogeneous environments. In the field, factors such as alternative hosts, intraguild interactions, plant volatiles, and spatial structure can strongly influence parasitoid searching efficiency and host acceptance (Colinet and Hance 2010, Thakur and Rawat 2014). Consequently, parasitism rates observed under controlled conditions may not directly translate to open-field performance.

Despite these constraints, our findings present encouraging evidence that certain commercially available parasitoid species—particularly *P. volucre* and *Aphi. matricariae*—are capable of parasitizing key orchard aphid pests and therefore warrant further investigation. Field trials will be essential to assess their efficacy under natural environmental variability, including their capacity for dispersal, establishment, and sustained population regulation. Such studies would allow a more accurate evaluation of whether these species can contribute to integrated pest management strategies aimed at reducing insecticide reliance and enhancing ecological sustainability in European fruit orchards.

## Supplementary Material

ieag023_Supplementary_Data
