# Supplementary material for: Evaluating biocontrol potential of 6 parasitoid species (Hymenoptera) on apple and cherry aphids (Hemiptera: Aphididae) using no-choice bioassays
Source: J Insect Sci. 2026 Mar 15;26(2):ieag023. doi: 10.1093/jisesa/ieag023 (PMC12989101; doi:10.1093/jisesa/ieag023)
Supplement: ieag023_Supplementary_Data [file ieag023_supplementary_data.zip › Supplementary material.docx]

# Supplementary Material

**Supplementary Figure S1 Caption.** Temperature (orange) and relative humidity (RH, blue) in the greenhouse during the rearing of M. cerasi and the incubation period of its parasitism bioassay (28th May–20th June). Daily fluctuations in temperature and RH were driven by natural light heating in the greenhouse. Temperature ranged from 18 to 45 °C, with a mean of approximately 24 °C, while RH varied between 19 and 85%, with a mean of 59%.

**Supplementary Figure S1 Alt text.** Line graph depicting temperature (T) in orange and relative humidity (RH) in blue, registered in the greenhouse during the rearing of M. cerasi. On the x-axis is the incubation period of its parasitism bioassay (from 28th May to 20th June). On the y-axis the ranges of the variables of T and RH, which share the same y-axis scale. Temperature fluctuates daily, ranging from 18 to 45 °C, with a mean of approximately 24 °C, while RH varied daily between 19 and 85%, with a mean of 59%.

**Supplementary Figure S2 Caption.** Morphological variation of ***Dysaphis plantaginea*** mummies produced by different parasitoid genera. Mummies resulting from Aphidius parasitism all shared the characteristic golden coloration and elongated oval shape. Aphelinus produced elongated, uniformly black mummies, whereas those formed by Ephedrus were round, black, and exhibited a distinctive adhesive belt along the ventral side. In contrast, Praon induced pale mummies elevated on a conspicuous whitish “cocoon-like” pedestal beneath the abdomen.

**Supplementary Figure S2 Alt text. Four-panelled figure showing four aphid mummies on green leaves. For each panel, a genus name is written as title: *Aphidius, Aphelinus, Ephedrus, Praon*. The pictures show the different morphological appearance of parasitised aphids (*Dysaphis plantaginea*).** Mummies resulting from Aphidius parasitism all shared the characteristic golden coloration and elongated oval shape. Aphelinus produced elongated, uniformly black mummies, whereas those formed by Ephedrus were round, black, and exhibited a distinctive adhesive belt along the ventral side. In contrast, Praon induced pale mummies elevated on a conspicuous whitish “cocoon-like” pedestal beneath the abdomen.

**Supplementary Figure S3 Caption.** Boxplot of tree health (cherry shoots or apple saplings) evaluations for each experiment. Different colours of jitter points represent the respective treatments shown in the legend. Tree health was assessed visually on a scale from 1 (poor condition) to 5 (optimal condition). Jitter shows no colour clustering, indicating no correlation between treatment and tree health variables (*M. cerasi* R^2^ = 0.04, *A. pomi* R^2^ < 0.01, *D. plantaginea* R^2^ = 0.06).

**Supplementary Figure S3 Alt text.** The figure shows tree-health measurements across three aphid–parasitoid experiments. Each experiment is represented by a single grey vertical boxplot: M. cerasi on the left, A. pomi in the center, and D. plantaginea on the right. The boxplots summarize overall tree-health scores within each experiment. Individual data points are overlaid as jittered dots and colored according to the parasitoid species used in each treatment, including the control. The points are spread horizontally to avoid overlap, illustrating the variability in tree health among replicates. The distribution of colored points within each experiment appears mixed rather than forming treatment-specific clusters, indicating that tree-health variation is not strongly associated with particular parasitoid treatments. A legend on the right identifies all treatments using color.

**Supplementary figure S4 Caption:** Numbers of live aphids, dead aphids, and mummies recorded across treatments in the *Myzus cerasi* bioassay. Boxplots show the distribution of counts for each response variable within each parasitoid treatment and the control. Points represent outliers. Live aphids (orange), dead aphids (green), and mummies (grey) are plotted on the same scale to illustrate treatment-specific differences in aphid mortality and parasitism outcomes. This assay was generally characterised by high natural mortality.

**Supplementary figure S4 Alt text:** Boxplot figure showing three sets of boxplots for the Myzus cerasi bioassay: live aphids, dead aphids, and mummies. Each set displays seven treatments on the x-axis: Control, Aphelinus abdominalis, Aphidius colemani, Aphidius ervi, Aphidius matricariae, Ephedrus cerasicola, and Praon volucre. The y-axis shows the number of individuals. Boxplots visualize the distribution of counts per treatment, and individual points represent outliers. Live aphids are shown in orange, dead aphids in green, and mummies in grey. All three response variables share the same y-axis scale.

**Supplementary figure S5 Caption:** Numbers of live aphids, dead aphids, and mummies recorded across treatments in the *Aphis pomi* bioassay. Boxplots show the distribution of counts for each response variable within each parasitoid treatment and the control. Points represent outliers. Live aphids (orange), dead aphids (green), and mummies (grey) are plotted on the same scale to illustrate treatment-specific differences in aphid mortality and parasitism outcomes. The control treatment was performed with one replicate. This assay was generally characterised by high aphid survival and medium natural mortality.

**Supplementary figure S5 Alt text:** Boxplot figure showing three sets of boxplots for the Aphis pomi bioassay: live aphids, dead aphids, and mummies. Each set displays seven treatments on the x-axis: Control, Aphelinus abdominalis, Aphidius colemani, Aphidius ervi, Aphidius matricariae, Ephedrus cerasicola, and Praon volucre. The y-axis shows the number of individuals. Boxplots visualize the distribution of counts per treatment, and individual points represent outliers. Live aphids are shown in orange, dead aphids in green, and mummies in grey. All three response variables share the same y-axis scale.

**Supplementary figure S6 Caption:** Numbers of live aphids, dead aphids, and mummies recorded across treatments in the *Dysaphis plantaginea* bioassay. Boxplots show the distribution of counts for each response variable within each parasitoid treatment and the control. Points represent outliers. Live aphids (orange), dead aphids (green), and mummies (grey) are plotted on the same scale to illustrate treatment-specific differences in aphid mortality and parasitism outcomes. This assay was generally characterised by low natural mortality.

**Supplementary figure S6 Alt text:** Boxplot figure showing three sets of boxplots for the Dysaphis plantaginea bioassay: live aphids, dead aphids, and mummies. Each set displays seven treatments on the x-axis: Control, Aphelinus abdominalis, Aphidius colemani, Aphidius ervi, Aphidius matricariae, Ephedrus cerasicola, and Praon volucre. The y-axis shows the number of individuals. Boxplots visualize the distribution of counts per treatment, and individual points represent outliers. Live aphids are shown in orange, dead aphids in green, and mummies in grey. All three response variables share the same y-axis scale.

**Supplementary Table S1:** Raw aphid counts. Minimum, maximum, mean and median number of mummies, alive aphids and dead aphids per treatment, across the three experiments.
