# Supplementary material for: Evaluating biocontrol potential of 6 parasitoid species (Hymenoptera) on apple and cherry aphids (Hemiptera: Aphididae) using no-choice bioassays
Source: J Insect Sci. 2026 Mar 15;26(2):ieag023. doi: 10.1093/jisesa/ieag023 (PMC12989101; doi:10.1093/jisesa/ieag023)
Supplement: ieag023_Supplementary_Data [file ieag023_supplementary_data.zip › Table S1.docx]

| **Experiment** | **Treatment** | **Avg mummies** | **Median mummies** | **Min mummies** | **Max mummies** | **Avg alive aphids** | **Median alive aphids** | **Min alive aphids** | **Max alive aphids** | **Avg dead aphids** | **Median dead aphids** | **Min dead aphids** | **Max dead aphids** |
| --- | --- | --- | --- | --- | --- | --- | --- | --- | --- | --- | --- | --- | --- |
| *Myzus cerasi* | Control | 0 | 0 | 0 | 0 | 18 | 0 | 0 | 80 | 71.1 | 78 | 8 | 137 |
|  | *Aphelinus abdominalis* | 7.4 | 2 | 0 | 43 | 8.8 | 0 | 0 | 67 | 39.6 | 34.5 | 25 | 56 |
|  | *Aphidius colemani* | 5 | 2 | 0 | 16 | 19.4 | 9 | 0 | 64 | 47.1 | 45.5 | 32 | 76 |
|  | *Aphidius ervi* | 1.8 | 0 | 0 | 12 | 15.2 | 1 | 0 | 89 | 20 | 16.5 | 6 | 45 |
|  | *Aphidius matracariae* | 14.6 | 5 | 2 | 65 | 5.2 | 1 | 0 | 29 | 21.2 | 22 | 5 | 45 |
|  | *Ephedrus cerasicola* | 24.9 | 9 | 0 | 89 | 12.1 | 5 | 1 | 39 | 35.5 | 37.5 | 8 | 72 |
|  | *Praon volucre* | 9.1 | 7.5 | 0 | 27 | 7.5 | 2 | 0 | 46 | 38.5 | 41.5 | 7 | 60 |
| *Aphis pomi* | Control | 0 | 0 | 0 | 0 | 27 | 27 | 27 | 27 | 2 | 2 | 2 | 2 |
|  | *Aphelinus abdominalis* | 2.2 | 2 | 1 | 4 | 17.3 | 16.5 | 5 | 32 | 4.3 | 3.5 | 0 | 11 |
|  | *Aphidius colemani* | 8.7 | 9.5 | 1 | 17 | 10 | 10.5 | 2 | 17 | 2.8 | 2 | 0 | 8 |
|  | *Aphidius ervi* | 3.7 | 3.5 | 0 | 7 | 11.7 | 12.5 | 3 | 23 | 7.3 | 6.5 | 0 | 18 |
|  | *Aphidius matracariae* | 8 | 6.5 | 2 | 20 | 17.8 | 17.5 | 5 | 34 | 2.5 | 0 | 0 | 9 |
|  | *Ephedrus cerasicola* | 0 | 0 | 0 | 0 | 29.2 | 31.5 | 17 | 41 | 0.8 | 0 | 0 | 3 |
|  | *Praon volucre* | 9 | 8 | 1 | 17 | 19.2 | 18.5 | 0 | 38 | 11.7 | 10.5 | 0 | 27 |
| *Dysaphis plantaginea* | Control | 0 | 0 | 0 | 0 | 147.5 | 93 | 19 | 400 | 1.8 | 0 | 0 | 11 |
|  | *Aphelinus abdominalis* | 12.5 | 7 | 1 | 31 | 2.5 | 0.5 | 0 | 10 | 18.8 | 27 | 0 | 30 |
|  | *Aphidius colemani* | 9.7 | 5 | 4 | 25 | 70.7 | 40.5 | 0 | 200 | 7.8 | 0 | 0 | 24 |
|  | *Aphidius ervi* | 20.3 | 17 | 4 | 45 | 5.2 | 1.5 | 0 | 15 | 12.2 | 13 | 0 | 25 |
|  | *Aphidius matracariae* | 28.8 | 23 | 0 | 56 | 30.8 | 35 | 0 | 50 | 5 | 0 | 0 | 30 |
|  | *Ephedrus cerasicola* | 26.5 | 24.5 | 0 | 64 | 114.2 | 102.5 | 20 | 200 | 0 | 0 | 0 | 0 |
|  | *Praon volucre* | 27 | 31.5 | 0 | 40 | 14 | 3 | 0 | 50 | 6.5 | 0 | 0 | 30 |
