# Supplementary figures and images for: Evaluating biocontrol potential of 6 parasitoid species (Hymenoptera) on apple and cherry aphids (Hemiptera: Aphididae) using no-choice bioassays
Source: J Insect Sci. 2026 Mar 15;26(2):ieag023. doi: 10.1093/jisesa/ieag023 (PMC12989101; doi:10.1093/jisesa/ieag023)

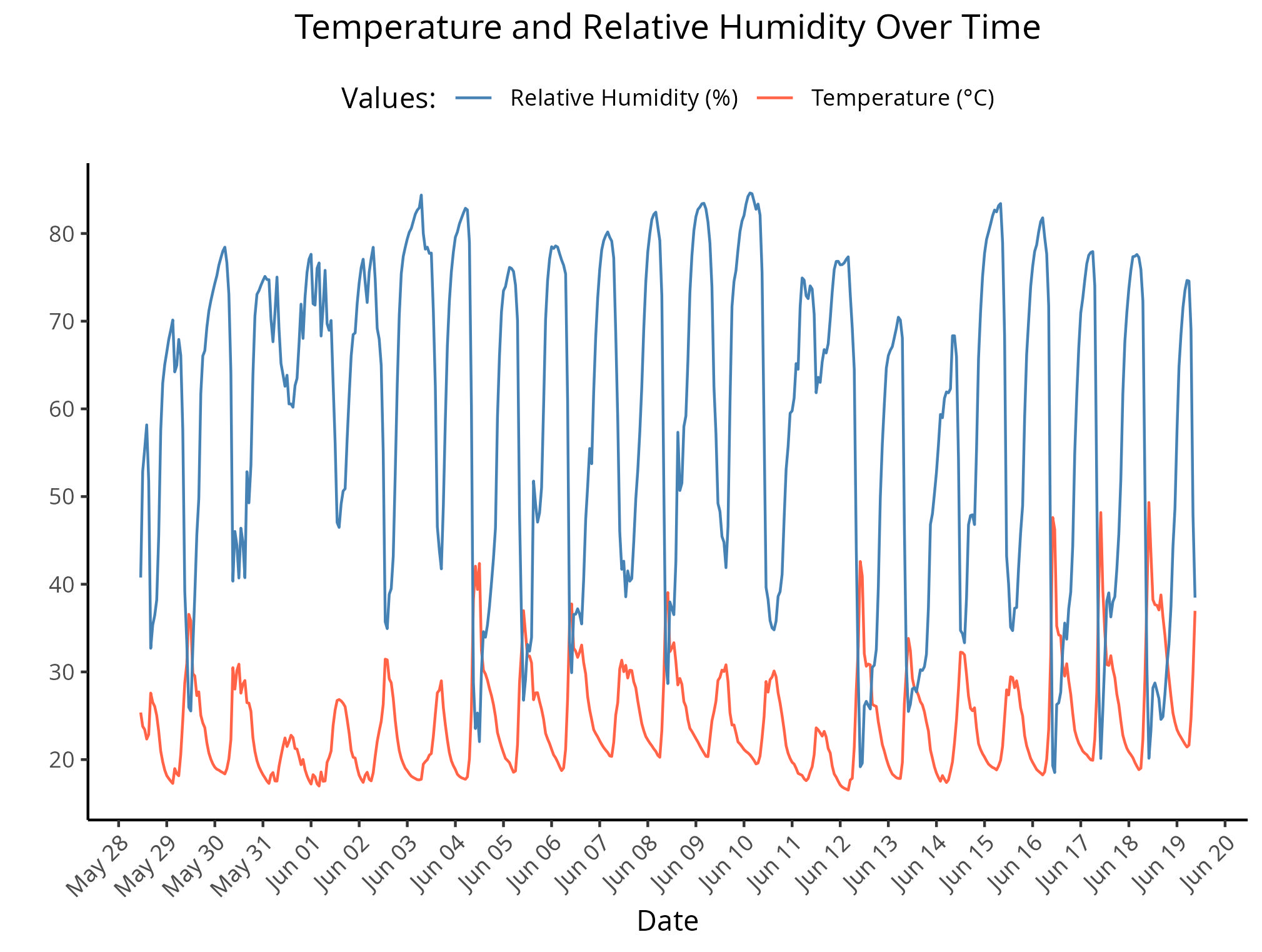

Supplement: ieag023_Supplementary_Data [file ieag023_supplementary_data.zip › S1.png]

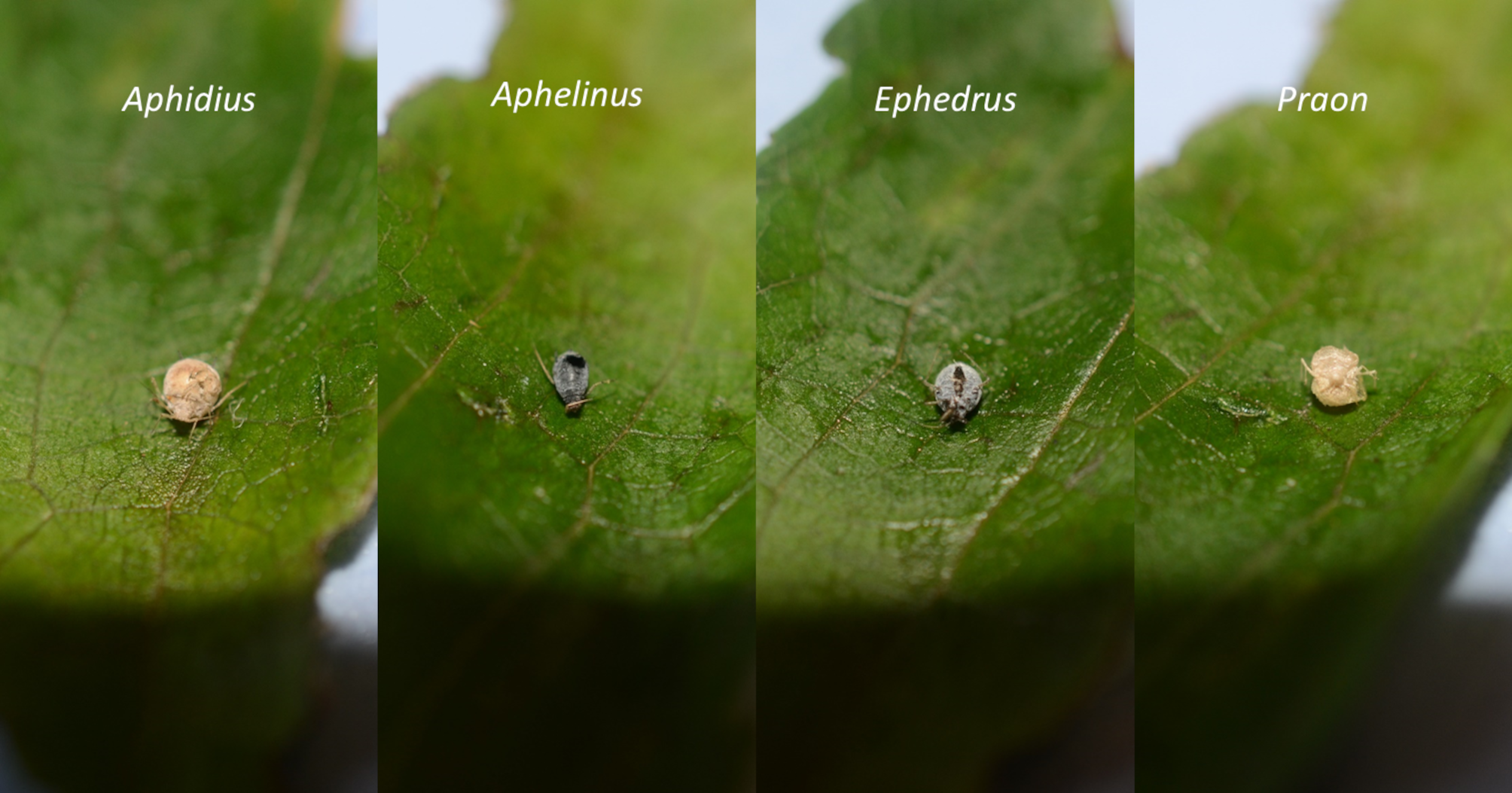

Supplement: ieag023_Supplementary_Data [file ieag023_supplementary_data.zip › S2.png]

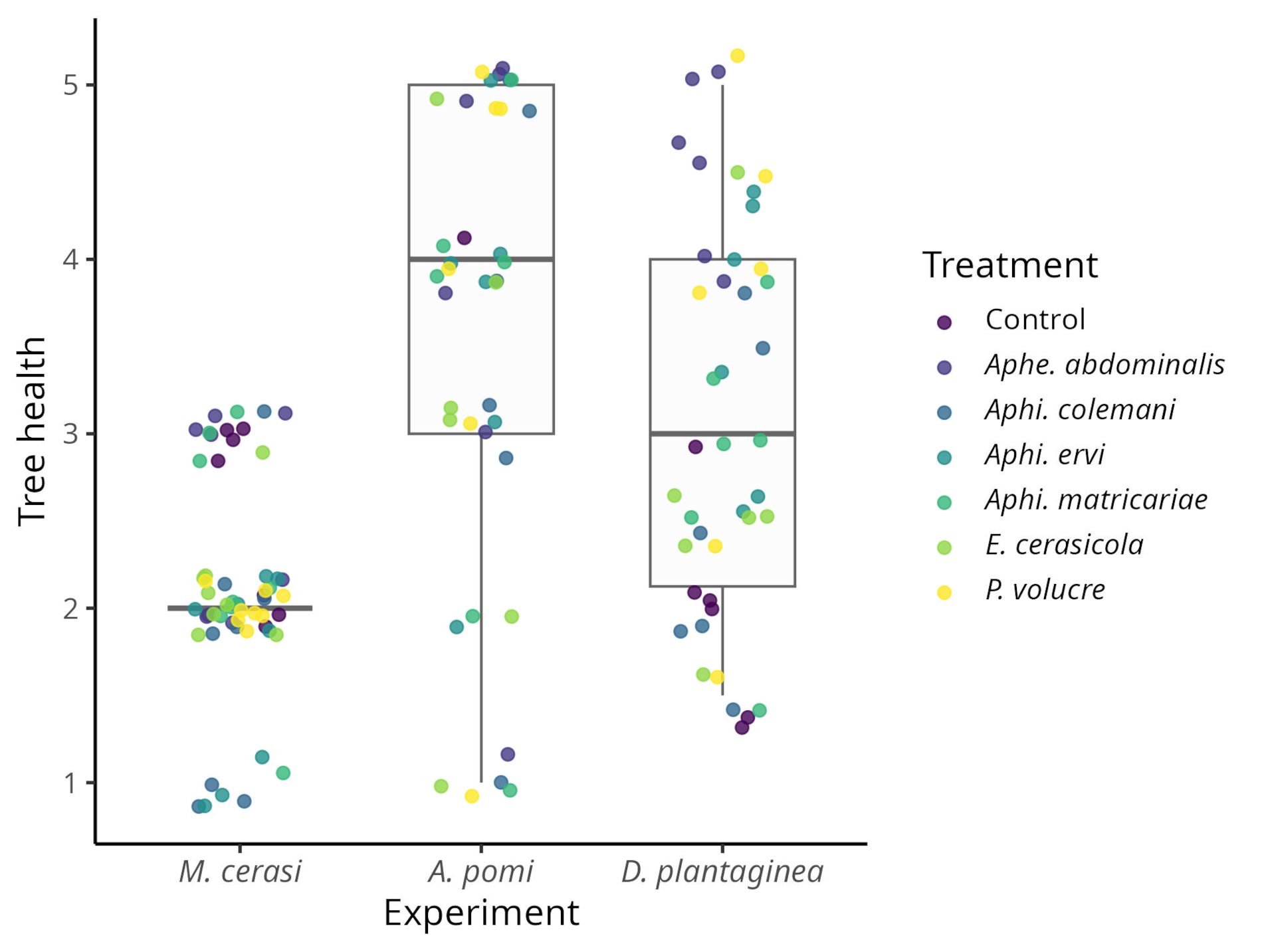

Supplement: ieag023_Supplementary_Data [file ieag023_supplementary_data.zip › S3.png]

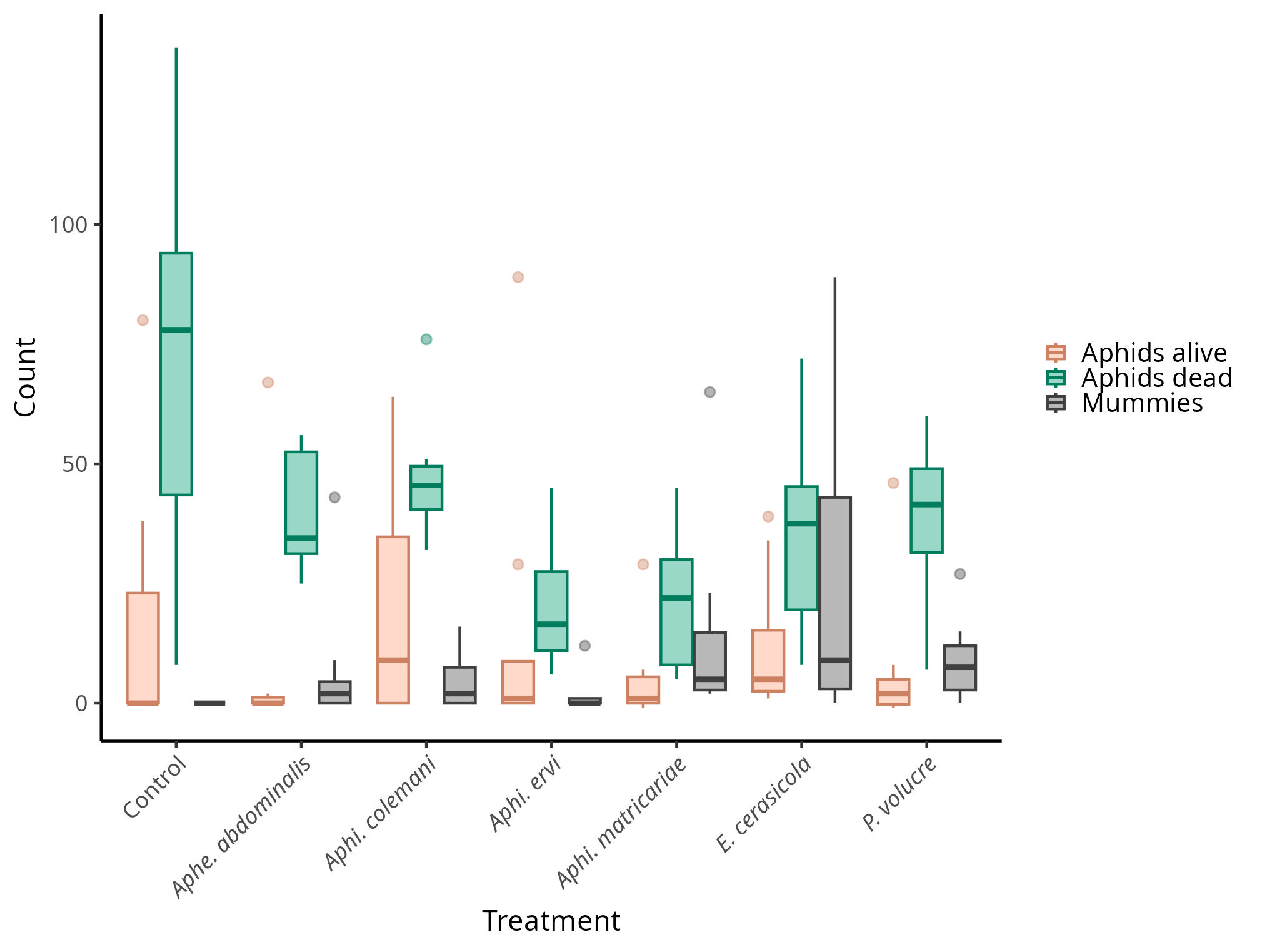

Supplement: ieag023_Supplementary_Data [file ieag023_supplementary_data.zip › S4.png]

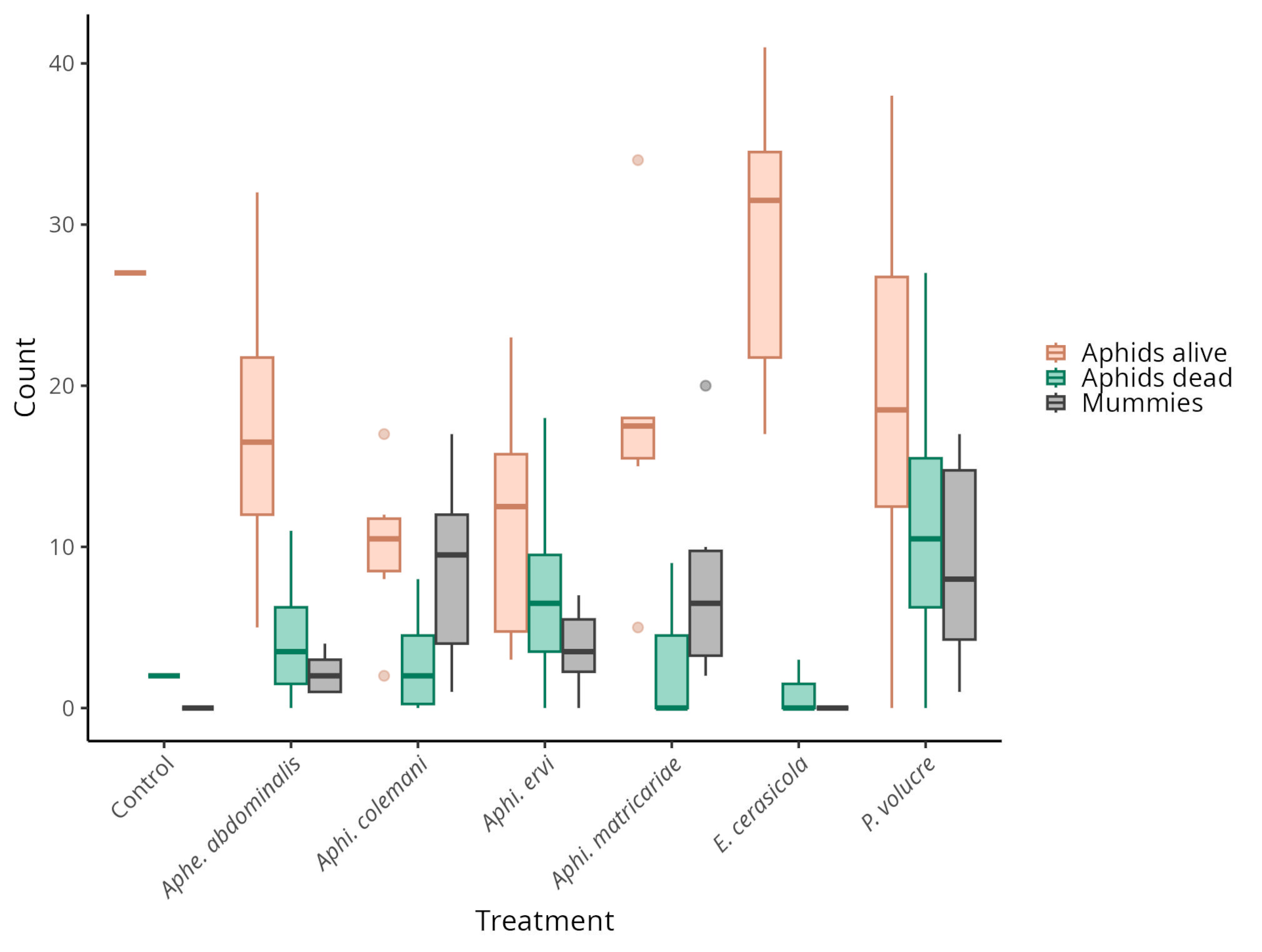

Supplement: ieag023_Supplementary_Data [file ieag023_supplementary_data.zip › S5.png]

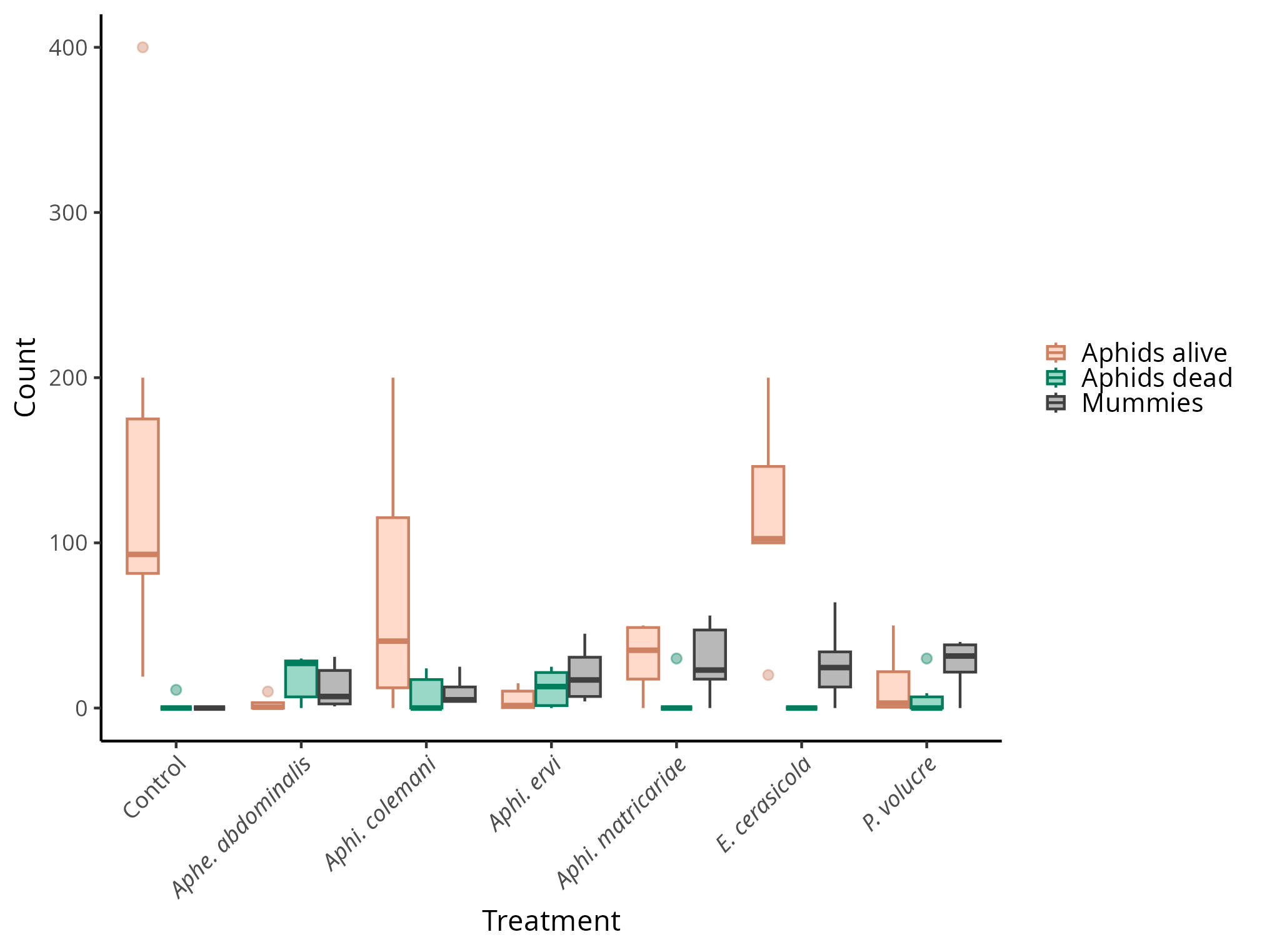

Supplement: ieag023_Supplementary_Data [file ieag023_supplementary_data.zip › S6.png]
